# Highly-efficient quantum memory for polarization qubits in a spatially-multiplexed cold atomic ensemble

**DOI:** 10.1038/s41467-017-02775-8

**Published:** 2018-01-25

**Authors:** Pierre Vernaz-Gris, Kun Huang, Mingtao Cao, Alexandra S. Sheremet, Julien Laurat

**Affiliations:** 1 Laboratoire Kastler Brossel, Sorbonne Université, CNRS, ENS-PSL Research University, Collège de France, 4 place Jussieu, 75005 Paris, France; 2 Centre for Quantum Computation and Communication Technology, Research School of Physics and Engineering, The Australian National University, Canberra, ACT 2601 Australia; 3 Present Address: Shanghai Key Laboratory of Modern Optical Systems, and Engineering Research Center of Optical Instruments and Systems (Ministry of Education), School of Optical Electrical and Computer Engineering, University of Shanghai for Science and Technology, Shanghai, 200093 China

## Abstract

Quantum memory for flying optical qubits is a key enabler for a wide range of applications in quantum information. A critical figure of merit is the overall storage and retrieval efficiency. So far, despite the recent achievements of efficient memories for light pulses, the storage of qubits has suffered from limited efficiency. Here we report on a quantum memory for polarization qubits that combines an average conditional fidelity above 99% and efficiency around 68%, thereby demonstrating a reversible qubit mapping where more information is retrieved than lost. The qubits are encoded with weak coherent states at the single-photon level and the memory is based on electromagnetically-induced transparency in an elongated laser-cooled ensemble of cesium atoms, spatially multiplexed for dual-rail storage. This implementation preserves high optical depth on both rails, without compromise between multiplexing and storage efficiency. Our work provides an efficient node for future tests of quantum network functionalities and advanced photonic circuits.

## Abstract (web summary)

Future quantum networks will require quantum memories with effective storage-and-retrieval capabilities. Here, the authors use electromagnetically-induced transparency in a high optical-depth, spatially-multiplexed cold atom ensemble to store and retrieve polarization qubits with high efficiency.

## Introduction

Quantum memories enabling the storage of an input photonic qubit and its later retrieval with a fidelity beating any classical device constitute essential components in quantum communication networks and optical quantum information processing^1,2^. Over the past years, storage of optical qubits has been demonstrated in a variety of physical platforms, including individual atoms in high-finesse cavities^3^, ion-doped crystals,^4–6^ and large ensembles of neutral atoms^7–9^. Quantum memories capable of storing qubits encoded into multiple degrees of freedom of light have also been achieved recently^10^.

The storage and retrieval efficiency of such devices is a stringent parameter for the envisioned applications^2,11–13^ and boosting this parameter has been a long-standing quest. This figure of merit is crucially important to reduce the entanglement distribution time in quantum repeater architectures and thereby develop scalable communication links^9,14,15^. It is also essential for increasing the success rate of gate operations^16^ or for building up iterative quantum state engineering schemes in optical quantum circuits^17^. A memory efficiency exceeding the important 50% threshold would as well enable protocols to perform in the no-cloning regime without post-selection^18^ or error correction for qubit losses in linear optics quantum computation^19^. However, to date, the highest storage and retrieval efficiencies achieved for qubits, independently of the photonic degrees of freedom, are below 30%^10,20–24^.

These limited values contrast with the recent progresses achieved in the demonstrations of optical memories. Ultra-high optical depths (OD) have indeed been obtained in laser-cooled elongated ensembles of neutral atoms^25,26^ and then used to realize high-efficiency single-mode optical storage^27,28^ based on long-lived collective excitations. However, the increase in OD, which is a strong prerequisite for large efficiency^29^, often comes at the expense of spatial multimode capacity as the atomic cloud is elongated along a direction and radially compressed. It results in a reduced transverse size that may render some strategies such as dual-rail storage arduous. Qubit storage with large efficiency remains thereby a challenging goal.

Here we demonstrate a faithful quantum memory for polarization qubits with a storage and retrieval efficiency close to 70%. Our realization is based on electromagnetically induced transparency (EIT) in a single spatially -multiplexed ensemble of cold cesium atoms featuring a large OD. The qubits are implemented using attenuated coherent states at the single-photon level. The reported efficiency approaches the maximal performance achievable on the D_2_ line used here, as shown by a comprehensive model that includes all the involved atomic transitions. Relative to previous works, this advance has been made possible by combining a high OD medium, efficient spatial multiplexing and low -noise operation.

## Results

### Preparation of high-OD cold atomic ensemble

**Fig. 1:**
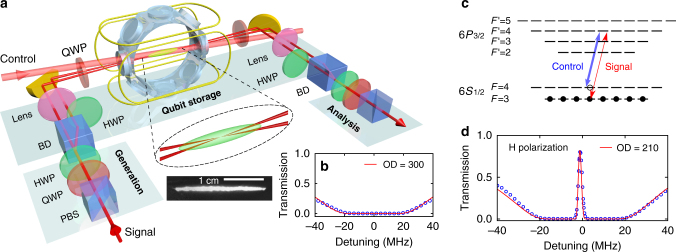
Quantum memory for polarization qubits in a multiplexed large OD atomic cloud. **a** A polarization qubit is encoded via a quarter (QWP) and a half wave plate (HWP) and converted into a dual-rail qubit with a beam displacer (BD). The orthogonally polarized beams, separated by 4 mm, are then mapped into an elongated ensemble of laser-cooled cesium atoms prepared in a 2D magneto-optical trap in a glass chamber. The spatial multiplexing is realized by focusing the two parallel paths into the 2.5-centimeter-long ensemble with a small crossing angle of 0.5° in order to preserve a large OD for each mode, an essential but challenging feature. A single control beam propagates with an angle of 1° relative to the signal modes in the plane of symmetry. **b** A large OD of 300 is obtained. The blue points correspond to the experimental data while the red solid line gives the theoretical fit. **c** Energy levels of the Cs D_2_ line involved in the EIT scheme. The atoms are prepared in *F* = 3 and populate all the Zeeman levels. Signal and control fields have the same circular polarization to avoid residual absorption. A comprehensive model is derived to take into account all the atomic levels, including the excited levels out of resonance. This model allows to understand the fundamental limits for storage and retrieval in such a setting, as described in the text. **d** Typical EIT spectrum as a function of the signal detuning when the control beam is kept on resonance. The red solid line corresponds to the full model

To obtain an ensemble with large OD, our experiment is based on an elongated 2D magneto-optical trap (MOT) of cesium atoms^25,26,30^. As sketched in Fig. 1a, the MOT relies on two pairs of rectangular-shaped coils and three retro-reflected trapping beams with a two-inch diameter and a total power of 350 mW. The resulting cigar-shaped ensemble has a length of 2.5 cm.

The experiment cycle is completed with a repetition rate of 20 Hz (see Supplementary Note 1). After a 37.5-millisecond-long MOT loading, the OD is further increased by linearly ramping up the magnetic field gradient in the transverse directions to radially compress the ensemble. For this purpose, the trapping coil current is increased from 4 to 16 A over 8 ms. After switching off the MOT coils, polarization gradient cooling is performed for 1.95 ms by ramping down the power of the trapping and repump beams following an exponential profile, while ramping up the detuning of the trapping beam from −17 to −107 MHz.

The atoms are prepared in the $\left| g \right\rangle = \left| {6S_{1/2},F = 3} \right\rangle$ state by turning off the repump light earlier than the trapping light, as well as sending an additional depumping light resonant on the $\left| s \right\rangle = \left| {6S_{1/2},F = 4} \right\rangle$ to $\left| e \right\rangle = \left| {6P_{3/2},F{\prime} = 4} \right\rangle$ transition. This transfer is required as no perfect EIT can be obtained in the absence of Zeeman optical pumping if $\left| s \right\rangle$ is used as the initial ground state. Finally, experiments are started 2 ms after the MOT coils are turned off to allow sufficient decay of the magnetic field. Three pairs of coils are used to compensate residual magnetic fields and to limit the inhomogeneous broadening to 50 kHz, as measured by microwave spectroscopy.

At the end of the preparation stage, the OD for a probe resonant to the $\left| g \right\rangle \to \left| e \right\rangle$ transition reaches a value of about 300, as shown in Fig. 1b. The temperature of the atoms is measured to be 20 μK with a time-of-flight technique.

### Realization of high-efficiency memory

Having prepared a large OD ensemble, we now turn to the memory protocol. The reversible mapping is based on EIT that enables the conversion of a signal photon into a long-lived collective excitation by dynamically changing the power of an auxiliary control field^31–34^. The signal and control beams, which are tightly phase locked (Supplementary Note 1), have waist diameters of 250 μm and 2 mm, respectively, and they intersect at the center of the MOT with a small angle of 1°. The control beam on the $\left| s \right\rangle \to \left| e \right\rangle$ transition has the same circular polarization as the signal, as shown in Fig. 1c, to avoid absorption in an atomic system involving various EIT channels due to the presence of Zeeman sublevels. Proper alignment for the light polarization is especially important for a high OD medium, as only a small residual fraction of OD would lead to a significant absorption for the signal. Figure 1d gives a typical EIT spectrum. A transmission close to 80% is obtained at large OD.

**Fig. 2:**
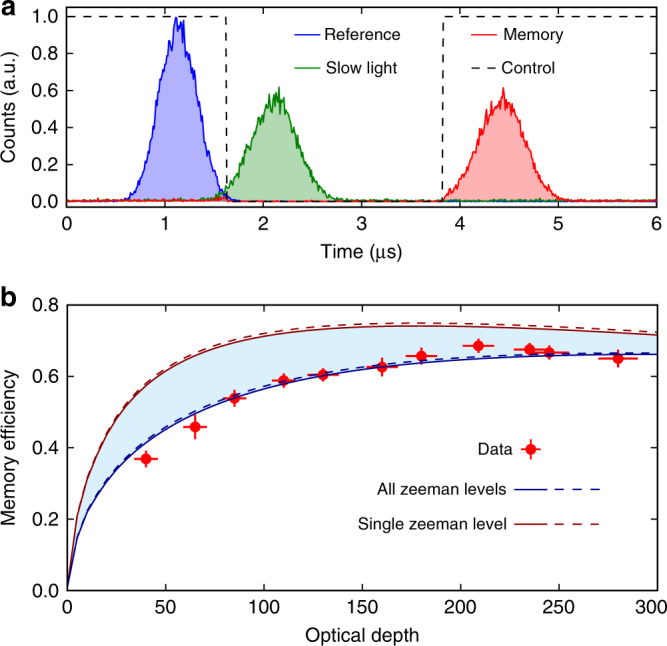
High-efficiency storage and retrieval. **a** Histogram of the photodetection counts. The blue-filled region gives the reference pulse without atoms while the green and red regions correspond to the slow and the stored-and-retrieved pulses, respectively. The black dashed line indicates the control intensity for the storage experiment. The memory efficiency reaches (69 ± 1)%. **b** Storage and retrieval efficiency as a function of the OD of the atomic cloud. The theoretical lines correspond to the two limiting cases with a single Zeeman level and all Zeeman levels, respectively. For each case, the solid lines correspond to an intrinsic ground-state decoherence estimated to *γ*_0_ = 10^−3^Γ, while the dashed lines correspond to the limiting case with *γ*_0_ = 0. Γ denotes the natural linewidth of the excited state. The errors are obtained from multiple independent measurements

To ensure negligible leakage during the storage process, the power of the control beam is chosen to provide a slow-light delay equal to twice the probe pulse duration when the control is continuously on. Figure 2a gives a single-photon level measurement of the slowed pulse and an example of a dynamic memory operation with a few microseconds storage. Before detection, the signal passes through a home-made atomic filter and a commercial lens-based cavity for spectral filtering (Quantaser FPE001A), with an overall rejection of 70 dB for the control field.

Thanks to the large OD achieved here, we could investigate the scaling behavior of the storage and retrieval efficiency. Figure 2b shows the efficiency as a function of the OD, which is varied by adjusting the power of the trapping beams during loading. At each value, the time delay is maintained constant by adapting the control beam power. As can be seen, the memory efficiency saturates at an OD of around 200 before decreasing. The maximal efficiency achieved here reaches (69 ± 1)%. This represents a record on the cesium D_2_ line and, more importantly, the highest achievable value in this configuration.

To understand this scaling and OD tradeoff, the complex level structure has to be taken into account. In the alkali-metal atoms, hyperfine interaction in the excited state indeed introduces several levels and EIT features can differ from the usual three-level Λ approximation, as previously studied in our group in various contexts^35–38^. Even for cold atoms, the off-resonance excitation of multiple excited levels can have a strong effect on the medium susceptibility. This is especially true for the D_2_ line of cesium atoms, for which the levels are only separated by 30 to 50Γ, where Γ denotes the natural linewidth of the excited state. These off-resonant excitations result in AC Stark shifts and effective additional ground state decoherence proportional to the control power. This decoherence rate limits the achievable transparency and therefore the storage efficiency at large ODs^28^. In addition, the atoms can be distributed in the ground state over many Zeeman sublevels, as it is the case in our experiment, and this configuration can lead to further inefficiency.

In Fig. 2b, experimental results are compared with a full model based on the Maxwell-Bloch equations and that takes into account the interaction of the probe and control field not only with all the excited levels but also with the Zeeman states (Supplementary Note 3). The theoretical lines correspond to the case with a single Zeeman level of the ground state $\left| g \right\rangle$, e.g. *m* = +3, and to the case with an equal population in all the Zeeman levels. As can be seen, the experimental results are in strong agreement with this second case. The efficiency would only be slightly increased to 75% by optical pumping, which is a challenging task for atomic ensembles with very large OD. The model also confirms that the intrinsic ground-state decoherence *γ*_0_ = (1.0 ± 0.5) × 10^−3^Γ estimated in our experiment is not the limiting factor for the memory performance.

### Implementation of a dual-rail memory

Next we present the extension of the setup to the qubit storage. The polarization mapping is implemented by using a dual-rail strategy in a single ensemble^39,40^. This method has been used in various implementations^10,41–43^, but the challenge was here to maintain a large OD and a strong noise cancellation despite this multiplexing in an elongated ensemble with a very reduced cross-section.

For this purpose, as illustrated in Fig. 1a, the signal beam passes through a beam displacer based on a birefringent calcite crystal (Thorlabs BD40) that provides a large 4-mm separation between the two orthogonally polarized beams. The two paths are then focused on the center of the MOT^44^ with a 500-mm focal length lens. Their transverse separation at the MOT edge is estimated to be 100 μm, which is much smaller than the 1-mm transverse size of the MOT. In contrast to the usual parallel scheme, i.e., without focusing the signal beam, here both beams cross the center of the MOT, enabling the attainment of a large and similar OD for the two paths. In the experiment, the OD is chosen to be around 200.

At the memory output, the two paths are recombined into a single spatial mode with a second beam displacer. The position of the second lens is optimized to obtain a visibility over 99% between these two paths, which is a critical step to achieve high fidelity. The two displacers form a passively stable Mach-Zehnder interferometer, where the relative phase is set to zero by adjusting the tilt of the second beam displacer.

### Highly efficient and faithful polarization qubit storage

**Fig. 3:**
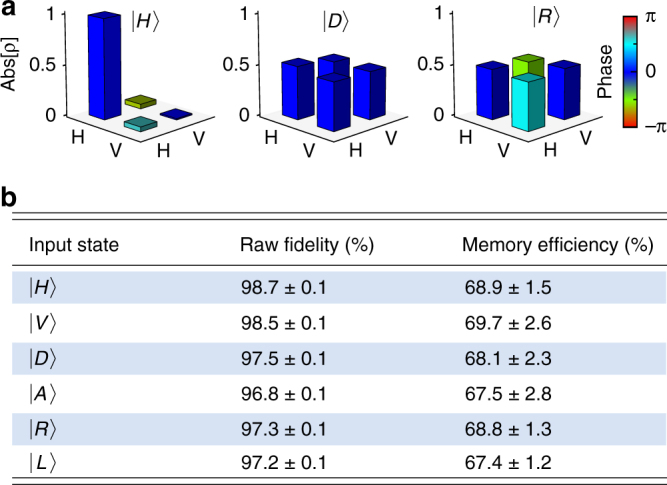
Quantum state tomography of the retrieved polarization qubits. **a** Reconstructed density matrices for the retrieved states after a 1.2-μs storage time. The height of the bar represents the absolute value while the color denotes the phase. No background has been subtracted. **b** Conditional fidelities and memory efficiencies for the set of six input qubits. The error bars for the fidelity are estimated by taking into account the statistical uncertainty of photon counts. The error for the efficiency is obtained from multiple measurements. The mean number of photons per pulse is $\bar n = 0.5$

We now proceed to the qubit storage in this dual-rail setting. Polarization qubits are implemented with weak coherent states with a mean photon number per pulse $\bar n$ = 0.5 and subsequently stored into the memory. The retrieved states are then characterized by usual quantum state tomography^45^. Figure 3a gives the reconstructed density matrices in the $\left\{ {\left| H \right\rangle ,\left| V \right\rangle } \right\}$ logical basis. From the measured matrices, one can estimate the conditional fidelity of the output states with the initially encoded state. The values for the complete set of inputs are listed in Fig. 3b. The average fidelity is 97.7 ± 0.8% and raises up to 99.5 ± 0.5% after correction for background noise that mainly comes from residual control leakage and detector dark counts. In the absence of an input signal, the background floor corresponds to 5 × 10^−4^ events per detection window.

**Fig. 4:**
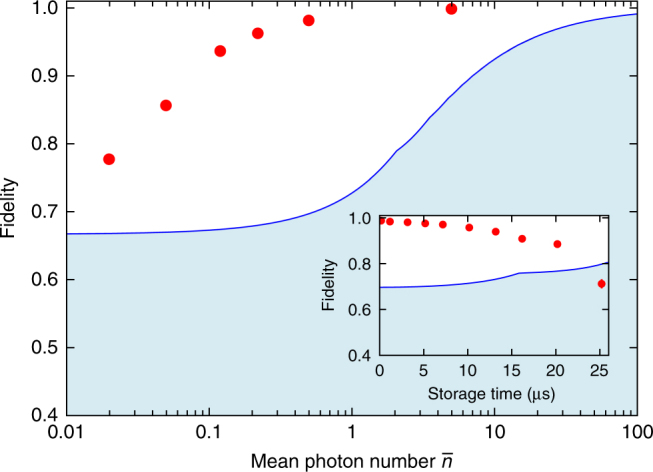
Storage and retrieval beyond classical benchmark. The fidelity is given as a function of the mean photon number per pulse $\bar n$, for a 1-μs storage time. The inset shows the fidelity as a function of the storage time, for a mean photon number $\bar n = 0.5$. No background correction has been applied. The blue solid line indicates the classical limit for the finite storage and retrieval efficiency and takes into account the Poissonian statistics of the weak coherent states. The error bars are smaller than the data points and are given by the standard deviations of fidelities for the set of stored states

In order to conclude about the quantum performance of the storage, we need to compare our fidelity with the maximal one achievable using a classical memory device, based for instance on the so-called measure and prepare strategy. It can be shown that the classical benchmark is given by a fidelity equal to (*N* + 1)/(*N* + 2) for a state containing *N* photons^46^, which is equal to 2/3 for the particular case of a single photon. In our case, the bound has to be modified to take into account the Poissonian statistics of the probe state and the finite memory efficiency, as done in refs. ^3,4^. Figure 4 presents the achieved fidelities as a function of the mean photon number per pulse $\bar n_{}^{}$. The measured fidelities are largely above the classical benchmark for $\bar n_{}^{}$ as low as 0.02. As shown in the inset of Fig. 4, the memory time for maintaining the quantum nature of the storage reaches more than 20 μs. In our experiment, the lifetime is mainly limited by the residual magnetic field along the elongated atomic cloud (Supplementary Note 2).

For these measurements, the average storage and retrieval efficiency reaches (68.5 ± 2)%, as detailed in Fig. 3b. This value is the highest efficiency reported so far for a reversible memory that demonstrates the quantum storage of photonic qubits. Moreover, we have shown that, because of the multiple level structure of the cesium D_2_ line, the achieved efficiency is the highest attainable in this configuration. Direct extension of our setup to the D_1_ line where excited levels are much more separated should enable to reach an efficiency above 90%^28^, although this has yet to be demonstrated in the quantum regime.

In our realization, the pulse duration of the qubits has been chosen around 400 ns to obtain the highest efficiency after one pulse-width delay. It corresponds to a memory bandwidth of a few MHz, as expected from EIT storage^2^. Single-photon sources with sub-MHz bandwidths have been demonstrated^47–49^ and can be adapted to our reported quantum memory.

## Discussion

In summary, we have demonstrated a highly efficient memory for optical qubits by successfully operating a large OD elongated atomic ensemble in a dual-rail configuration. This combination enables the reversible mapping of arbitrary polarization states not only with fidelities well above the classical benchmark but also with an overall storage and retrieval efficiency close to 70%. This value represents the highest efficiency to date for the storage and readout of optical qubits in any physical platform and is more than double of the previously -reported values. It also outperforms the important 50% threshold required to beat the no-cloning limit without post-selection.

Besides the aforementioned network architecture scalability and potential loss-tolerant schemes, the achieved efficiency opens the way to tests of advanced quantum networking tasks where the storage node efficiency plays a critical role, such as in certification protocols or unforgeable quantum money^50,51^. Moreover, the designed platform is directly compatible with recent works based on spatially -structured photons and multiple degree of freedom storage^10^ and can now yield very efficient realizations to boost high-capacity network channels.

### Data availability

The data that support the findings of this study are available from the corresponding authors on request.

## Electronic supplementary material

Supplementary information

## References

[1] Lvovsky AI, Sanders BC, Tittel W (2009). Optical quantum memory. Nat. Photon..

[2] Heshami K (2016). Quantum memories: emerging applications and recent advances. J. Mod. Opt..

[3] Specht HP (2011). A single-atom quantum memory. Nature.

[4] Gündogan M, Lendingham PM, Almasi A, Cristiani M, de Riedmatten H (2012). Quantum storage of photonic polarization qubit in a solid. Phys. Rev. Lett..

[5] Clausen C, Bussières F, Afzelius M, Gisin N (2012). Quantum storage of heralded polarization qubits in birefringent and anisotropically absorbing materials. Phys. Rev. Lett..

[6] Zhou ZQ, Lin WB, Yang M, Lin CF, Guo GC (2012). Realization of reliable solid-state quantum memory for photonic polarization qubit. Phys. Rev. Lett..

[7] Lukin MD (2003). Colloquium: trapping and manipulating photon states in atomic ensembles. Rev. Mod. Phys..

[8] Hammerer K, Sørensen AS, Polzik ES (2010). Quantum interface between light and atomic ensembles. Rev. Mod. Phys..

[9] Sangouard S, N C, de Riedmatten H, Gisin N (2011). Quantum repeaters based on atomic ensembles and linear optics. Rev. Mod. Phys..

[10] Parigi V (2015). Storage and retrieval of vector beams of light in a multiple-degree-of-freedom quantum memory. Nat. Commun..

[11] Kimble HJ (2008). The quantum internet. Nature.

[12] Walmsley IA (2015). Quantum optics: science and technology in a new light. Science.

[13] Bussières F (2013). Prospective applications of optical quantum memories. J. Mod. Opt..

[14] Briegel HJ, Dür W, Cirac JI, Zoller P (1998). Quantum repeaters: The role of imperfect local operations in quantum communication. Phys. Rev. Lett..

[15] Muralidharan. S (2016). Optimal architectures for long distance quantum communication. Sci. Rep..

[16] Chen W (2013). All-optical switch and transistor gated by one stored photon. Science.

[17] Felinto D (2006). Conditional control of the quantum states of remote atomic memories for quantum networking. Nat. Phys..

[18] Grosshans F, Grangier P (2001). Quantum cloning and teleportation criteria for continuous quantum variables. Phys. Rev. A..

[19] Varnava M, Browne D, Rudolph T (2006). Loss tolerance in one-way quantum computation via counterfactual error correction. Phys. Rev. Lett..

[20] Clausen C (2011). Quantum storage of photonic entanglement in a crystal. Nature.

[21] Lettner M (2011). Remote Entanglement between a Single Atom and a Bose-Einstein Condensate. Phys. Rev. Lett..

[22] Kalb N, Reiserer A, Ritter S, Rempe G (2015). Heralded storage of a photonic quantum bit in a single atom. Phys. Rev. Lett..

[23] Ding DS (2015). Raman quantum memory of photonic polarized entanglement. Nat. Photon..

[24] Zhou ZQ (2015). Quantum storage of three-dimensional orbital-angular-momentum entanglement in a crystal. Phys. Rev. Lett..

[25] Sparkes BM (2013). Gradient echo memory in an ultra-high optical depth cold atomic ensemble. New J. Phys..

[26] Hsiao YF, Chen HS, Tsai PJ, Chen YC (2014). Cold atomic media with ultrahigh optical depths. Phys. Rev. A..

[27] Cho YW (2016). Highly efficient optical quantum memory with long coherence time in cold atoms. Optica.

[28] Hsiao, Y.-F. et al. EIT-based photonic memory with near-unity storage efficiency. Preprint at: http://arXiv.org/abs/1605.08519 (2016).

[29] Gorshkov AV, André A, Fleischhauer M, Sørensen AS, Lukin MD (2007). Universal approach to optimal photon storage in atomic media. Phys. Rev. Lett..

[30] Zhang S (2012). A dark-line two-dimensional magneto-optical trap of Rb atoms with high optical depth. Rev. Sci. Instrum..

[31] Liu C, Dutton Z, Behroozi CH, Hau LV (2001). Observation of coherent optical information storage in an atomic medium using halted light pulses. Nature.

[32] Chanelière T (2005). Storage and retrieval of single photons transmitted between remote quantum memories. Nature.

[33] Eisaman MD (2005). Electromagnetically induced transparency with tunable single-photon pulses. Nature.

[34] Choi KS, Deng H, Laurat J, Kimble HJ (2008). Mapping photonic entanglement into and out of a quantum memory. Nature.

[35] Sheremet AS (2010). Quantum memory for light via a stimulated off-resonant raman process: beyond the three-level Λ-scheme approximation. Phys. Rev. A.

[36] Mishina OS (2011). Electromagnetically induced transparency in an inhomogeneously broadened Λ transition with multiple excited levels. Phys. Rev. A.

[37] Scherman M, Mishina OS, Lombardi P, Giacobino E, Laurat J (2012). Enhancing electromagnetically-induced transparency in a multilevel broadened medium. Opt. Express.

[38] Giner L (2013). Experimental investigation of the transition between Autler-Townes splitting and electromagnetically-induced-transparency models. Phys. Rev. A.

[39] Matsukevich DN, Kuzmich A (2004). Quantum state transfer between matter and light. Science.

[40] Chou CW (2007). Functional quantum nodes for entanglement distribution over scalable quantum networks. Science.

[41] Laurat J, Choi KS, Deng H, Chou CW, Kimble HJ (2007). Heralded entanglement between atomic ensembles: preparation, decoherence and scaling. Phys. Rev. Lett..

[42] England DG (2012). High-fidelity polarization storage in a gigahertz bandwidth quantum memory. J. Phys. B..

[43] Kupchak C (2014). Room-temperature single-photon level memory for polarization states. Sci. Rep..

[44] Ding DS (2016). Entanglement between low- and high-lying atomic spin waves. Phys. Rev. A.

[45] James DFV, Kwiat PG, Munro WJ, White AG (2001). Measurement of qubits. Phys. Rev. A.

[46] Massar S, Popescu S (1995). Optimal extraction of information from finite quantum ensembles. Phys. Rev. Lett..

[47] Du S, Kolchin P, Belthangady C, Yin GY, Harris SE (2008). Subnatural linewidth biphotons with controllable temporal length. Phys. Rev. Lett..

[48] Fekete J, Rieländer D, Cristiani M, de Riedmatten H (2013). Ultranarrow-band photon-pair source compatible with solid state quantum memories and telecommunication networks. Phys. Rev. Lett..

[49] Rambach M, Nikolova A, Weinhold TJ, White AG (2016). Sub-megahertz linewidth single photon source. APL Photon..

[50] Bartkiewicz K (2017). Experimental quantum forgery of quantum optical money. NPJ Quant. Inf..

[51] Bozzio, M. et al. Experimental demonstration of practical unforgeable quantum money. Preprint at: http://arxiv.org/abs/1705.01428 (2017).

