# Supplementary material for: Highly-efficient quantum memory for polarization qubits in a spatially-multiplexed cold atomic ensemble
Source: Nat Commun. 2018 Jan 25;9:363. doi: 10.1038/s41467-017-02775-8 (PMC5785556; doi:10.1038/s41467-017-02775-8)
Supplement: Supplementary file 1 — Supplementary information [file 41467_2017_2775_MOESM1_ESM.pdf]

Supplementary Note 1: Laser sources and experimental timing

Two main laser sources are used in the experiment. A Ti:sapphire laser (MSquared, SolsTiS) is stabilized on a reference cavity and frequency-locked via saturated absorption. It is used for seeding a tapered amplifier (Toptica Photonics, BoosTA) to deliver 350 mW for trapping beams. This laser source is also used for the control beam in the EIT protocol. The second laser source is an external-cavity diode (Toptica Photonics, DL Pro). It provides the 10-mW repumper and is also used for the probe. The two laser sources are phase-locked at the cesium hyperfine splitting frequency (Vescent Photonics, D2-135). Frequency tunings are realized via acousto-optical modulators in double-pass configuration.

The timing is shown in Supplementary Figure 1. The experiment is performed at a repetition rate of 20 Hz. The two pairs of coils (dimensions 270 mm × 110 mm) are driven by two independent power supplies (Delta Elektronika, SM52-30). After a trap loading phase of 37.5 ms, the atomic cloud is compressed by linearly ramping the current supply from 4 A to 16 A. The trapping magnetic field is then turned off ($t = 0$) via two home-made electronic switches. A preparation phase follows, with the trapping and repump powers ramped down while the trapping detuning is increased from -17 MHz to -107 MHz in 1 ms. Before the end of the preparation process, a 950-μs resonant (F=4 → F'=4) pulse and a 10-MHz red-detuned trapping beam are sent together to transfer the atoms to the F=3 ground state. The trapping coils current is sinked with an exponential decay time of about 50 μs. However, the induced eddy currents of the surrounding metallic components have a longer decay time. To reduce their perturbations, the residual magnetic field cancellation via three pairs of coils is optimized 2 ms later after the switching. Memory operations are then performed during a period of 1 ms. Depending on the storage time, this interval is split into 25 to 100 repetitions. Photons are detected by a single avalanche photodiode (SPCM-AQR-14-FC) and recorded with a FPGA-based digitizer with a time resolution of 10 ns. For each polarization projection, 10^5 memory sequences are accumulated.

Supplementary Note 2: Memory lifetime and decoherence

Three decoherence mechanisms can be evaluated independently. First, the atomic motion related to the finite temperature results in a possible loss of the atoms from the interaction area. The thermal velocity of an atom of mass m at a temperature T is given by $v = \sqrt{k_B T/m}$. The transit time can therefore be estimated by $\tau_1 = D/v$ with $D \simeq 250$ μm the diameter of the probe beam. With a temperature T estimated at 20 μK by a time-of-flight measurement, the corresponding transit time is $\tau_1 = 7$ ms. This is not a limiting factor in our experiment.

The two other decoherence contributions come from the dephasing of the collective excitation. The first source of possible dephasing is the so-called motional dephasing due to the strong angular dependence of EIT [1, 2]. In the experiment, we used indeed an off-axis configuration and the control and probe beams are overlapped with an angle

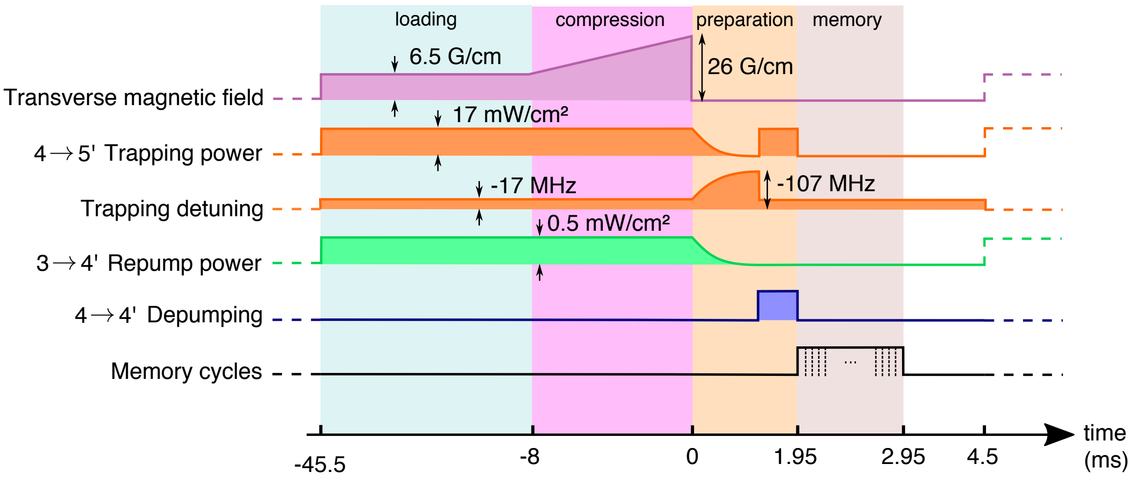

Supplementary Figure 1: Timing diagram of the experiment.

$\theta \simeq 1°$. The resulting lifetime is then given by:

$$\tau_2 = \frac{\lambda}{2\pi \sin\theta}\sqrt{\frac{m}{k_B T}}. \quad (1)$$

In our case, this expression leads to a decay time $\tau_2 = 220$ µs. The second dephasing process is caused by residual magnetic fields which result in an atom-dependent Larmor precession. The magnetic field is compensated via three pairs of coils and the inhomogeneous broadening in the ground state is therefore limited to around 50 kHz, as measured via microwave spectroscopy. By assuming a gradient of magnetic field equal to 8 mG/cm along the length of the ensemble $L = 2.5$ cm and a Gaussian atomic distribution, the expected time constant is $\tau_3 = 15$ µs [3]. This dephasing is the main decoherence source in our experiment.

Supplementary Figure 2 gives the retrieval efficiency as a function of the storage duration. The solid line corresponds to the full model derived in the following section.

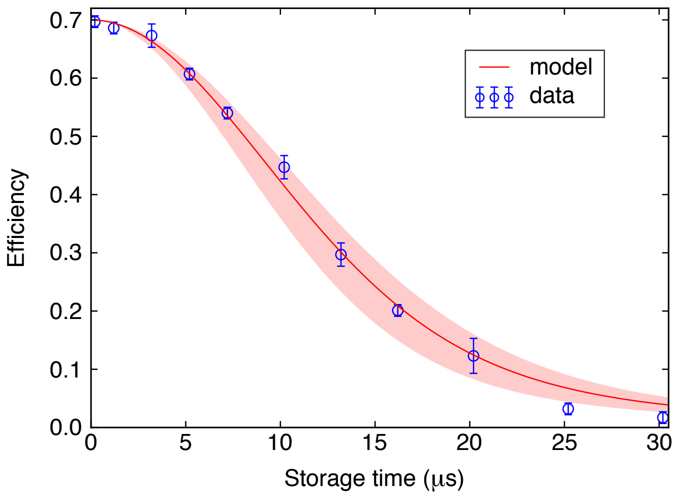

Supplementary Figure 2: Retrieval efficiency as a function of the storage time. The blue points give the experimental measurements while the red solid line corresponds to the full model explained in Supplementary Note 3, with a measured OD equal to 200 and a 50 kHz inhomogeneous broadening. The shaded area corresponds to an uncertainty on the residual magnetic field about ± 1 mG.cm^{-1}. Errors were estimated assuming Poissonian statistics.

Supplementary Note 3: Multi-level theoretical model for light storage and retrieval

In our theoretical description, we consider the interaction of the probe and control fields with the full D_2-line structure of ^{133}Cs atoms. The influence of the multi-level structure in alkali-metal atoms, beyond the three-level Λ approximation or double-Λ model, was studied theoretically and experimentally in our group in various contexts, e.g. electromagnetically-induced transparency (EIT) with broadened transitions [4, 5], Raman configuration [6] or EIT/ATS transition with cold atoms [7]. Even for cold atoms, the off-resonance excitation of multiple excited levels can have a strong effect on the medium susceptibility and therefore on the memory efficiency. In addition to all the excited levels, we also include here the Zeeman sublevels to compare implementations with and without optical pumping.

A. Level scheme: multiple excited levels and Zeeman sublevels

The level scheme is shown in Supplementary Figure 3. Atoms equally populate the Zeeman sublevels $|g_m\rangle = |6S_{1/2}, F = 3, m\rangle$, which are coupled to the excited states $|e_{F',n}\rangle = |6P_{3/2}, F' = 2, 3, 4, n = m + 1\rangle$ by a weak σ^+-polarized probe field. The σ^+-polarized control field couples the second ground state $|s_m\rangle = |6S_{1/2}, F = 4, m\rangle$ with the excited levels $|e_{F',n}\rangle = |6P_{3/2}, F' = 3, 4, 5, n = m + 1\rangle$. The atomic medium is optically thick for the probe field. Such polarization scheme with atoms initially in $F_g = 3$ provides interaction of the probe with the atomic ensemble without residual absorption in the absence of optical pumping. The detunings of the probe field with frequency ω_p and the control field with frequency ω_c from the atomic transitions $|g_m\rangle \to |e_{F'=4,n}\rangle$ and $|s_m\rangle \to |e_{F'=4,n}\rangle$ in the absence of a magnetic field are noted as $\Delta_p = \omega_p - \omega_{e_{F',n} g_m}$ and $\Delta_c = \omega_c - \omega_{e_{F',n} s_m}$ respectively.

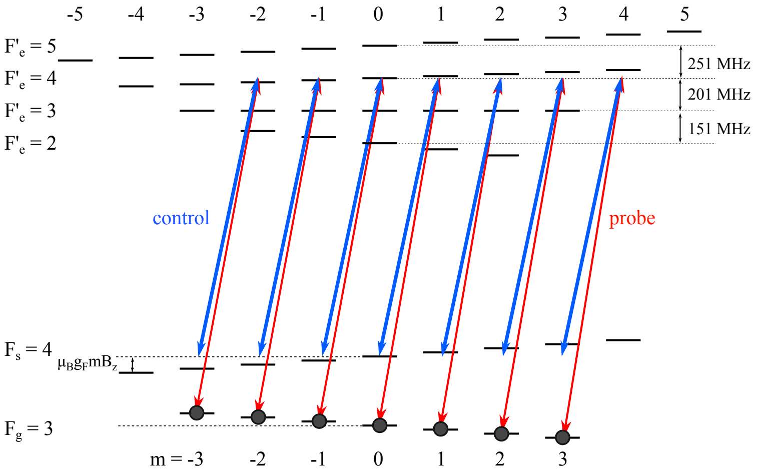

Supplementary Figure 3: Level scheme for [133]Cs D_2-line. The σ^+-polarized control field couples the $|F_s = 4, m\rangle \to |F'_e = 4, m+1\rangle$ transitions, while the σ^+-polarized probe field is scanned near resonance with $|F_g = 3, m\rangle \to |F'_e = 4, m+1\rangle$ transitions.

B. Residual magnetic field

In this model, we also include a residual magnetic field, which is the main decoherence source in our implementation. We consider a magnetic field gradient along the z axis with $B(z) = B_0 z$. This magnetic field $B(z)$ results in the splitting of Zeeman sublevels in the ground and excited states and leads to additional detuning for the probe and control fields from magnetically-insensitive transitions:

$$\delta_m^{(p,c)}(z) = \frac{\mu_B \left[m g_{F_{g,s}} - (m+1) g_{F'_e} \right] B(z)}{\hbar}, \tag{2}$$

where μ_B is the Bohr magneton and $g_{F_g}, g_{F_s}, g_{F'_e}$ are the hyperfine Landé factors for F_g, F_s and F'_e respectively.

C. Optical Bloch equations

The atomic system evolution is described by:

$$\frac{d\hat{\rho}}{dt} = \frac{i}{\hbar}\left[\hat{\rho}, \hat{H}\right] - \hat{\Gamma}\hat{\rho}. \tag{3}$$

Here $\hat{\Gamma}$ is a relaxation operator describing the radiative decay rate of the excited states and the decoherence processes in the ground state.

The Hamiltonian of the system can be written in the following form:

$$\hat{H} = \hat{H}_0 + \hat{V} = \hat{H}_{\text{atom}} + \hat{H}_{\text{field}} + \hat{V}_E + \hat{V}_B, \tag{4}$$

where the non-perturbative part of the Hamiltonian $\hat{H}_0$ is given by the sum of the free Hamiltonian operators of the atoms $\hat{H}_{\text{atom}}$ and the electromagnetic field $\hat{H}_{\text{field}}$. The dipole interaction part is written in the rotating-wave approximation and consists of the dipole interactions between the atom and the probe and control fields:

$$\hat{V}_E = \hat{V}_p + \hat{V}_c, \tag{5}$$

where

$$\hat{V}_p = -\sum_{m=-3}^{3} \sum_{F'=2,3,4} d_{e_{F',m+1} g_m} |e_{F',m+1}\rangle\langle g_m| E_p^{(+)} + H.c. \tag{6}$$

and

$$\hat{V}_c = -\sum_{m=-3}^{3} \sum_{F'=3,4,5} d_{e_{F',m+1} s_m} |e_{F',m+1}\rangle\langle s_m| E_c^{(+)} + H.c. \tag{7}$$

In the interaction picture, the positive frequency components of the electromagnetic field for the probe and the control modes are $E_p^{(+)} = \epsilon_p \cdot e^{-i\omega_p t}$ and $E_c^{(+)} = \epsilon_c \cdot e^{-i\omega_c t}$ respectively, $d_{ij} = \langle i|\hat{\mathbf{d}}|j\rangle$ is the electric dipole moment of the atom between levels i and j.

The optical Bloch equations can be written for slowly-varying amplitudes of the optical coherences $\sigma_{e_{F',m+1}g_m} = \rho_{e_{F',m+1}g_m} e^{i\omega_p t}$ and $\sigma_{s_m g_m} = \rho_{s_m g_m} e^{i(\omega_p - \omega_c)t}$ and solved following the approach developed in [4] as:

$$\begin{aligned}
\frac{d\sigma_{e_{F',m+1}g_m}(z)}{dt} &= i\left(\Delta_p + \delta_m^{(p)}(z) - \omega_{e_{F',m+1}e_{F'=4,m+1}}(z) + i\Gamma/2\right)\sigma_{e_{F',m+1}g_m}(z) \\
&\quad + i\left(\frac{\Omega^{(p)}_{e_{F',m+1}g_m}}{2}\rho_{g_m g_m} + \frac{\Omega^{(c)}_{e_{F',m+1}s_m}}{2}\sigma_{s_m g_m}(z)\right) \\
\frac{d\sigma_{s_m g_m}(z)}{dt} &= i\left((\Delta_p + \delta_m^{(p)}(z)) - (\Delta_c + \delta_m^{(c)}(z)) + i\gamma_0\right)\sigma_{s_m g_m}(z) + i\sum_{F'=3,4,5}\frac{\Omega^{(c)}_{s_m e_{F',m+1}}}{2}\sigma_{e_{F',m+1}g_m}(z)
\end{aligned} \tag{8}$$

where Γ is the excited state decay rate, γ_0 is the ground state decoherence, $\hbar \cdot \omega_{e_{F',m+1}e_{F'=4,m+1}}(z)$ is the energy difference between the hyperfine levels $|F'_e, m+1\rangle$ and $|F'_e = 4, m+1\rangle$ of the excited state at position z, $\Omega^{(p)} = 2\mathbf{d}\cdot\mathbf{E}_p/\hbar$ and $\Omega^{(c)} = 2\mathbf{d}\cdot\mathbf{E}_c/\hbar$ are the probe and control field Rabi frequencies respectively. The system of equations (8) is solved by taking the Fourier transform of the atomic coherences $\tilde{\sigma}_{ij}(\omega, z) = \int_{-\infty}^{\infty}\sigma_{ij}(t,z)e^{i\omega t}dt$.

The linear response of the atomic system to a weak probe field can then be described by the susceptibility given by the following expression:

$$\chi(\omega, z, \Delta_p, \Delta_c) = -\sum_{m=-3}^{3}\sum_{F'=2,3,4} n_0(z) d_{g_m e_{F',m+1}} \tilde{\sigma}_{e_{F',m+1}g_m}(\omega, z), \tag{9}$$

where $\tilde{\sigma}_{e_{F',m+1}g_m}(\omega, z)$ are the solutions for the slowly-varying amplitudes of the optical coherences between the ground state $|g_m\rangle$ and the excited state $|e_{F',m+1}\rangle$ addressed by the probe field. We assume an atomic cloud with a Gaussian distribution of the atomic density $n_0(z) = n_0 e^{-4z^2/L^2}$, where L is the length of the atomic medium.

D. Stark shift and additional ground state decoherence due to the excited levels

The non-resonant coupling of the control field with the excited states $|F'_e = 3, 5\rangle$ results in a significant modification of the atomic response compared to the standard Λ-scheme approximation [4]. These couplings result in Stark shifts and an additional effective ground state decoherence:

$$\begin{aligned}
\Delta_p^{(\text{eff})} &= \Delta_p + \sum_{m=-3}^{3}\sum_{F'=3,5}\frac{|\Omega^{(c)}_{e_{F',m+1}s_m}|^2/4}{\omega_{e_{F',m+1}e_{F'=4,m+1}}}, \\
\gamma_0^{(\text{eff})} &= \gamma_0 + \sum_{m=-3}^{3}\sum_{F'=3,5}\frac{|\Omega^{(c)}_{e_{F',m+1}s_m}|^2/4}{\omega^2_{e_{F',m+1}e_{F'=4,m+1}}}\frac{\Gamma}{2}.
\end{aligned} \tag{10}$$

Importantly, these additional terms depend on the power of the control beam. Recently these contributions were also described in [8] for N-type four-level model in the context of optical memories with atoms optically pumped to one Zeeman sublevel. In contrast, the present model includes not only the several excited levels but also the Zeeman sublevels, as they are involved in our experimental realization.

E. Loss during the pulse propagation in the medium

The loss during the pulse propagation in the medium gives the upper bound for the memory efficiency. This propagation can be described by the standard macroscopic Maxwell equation:

$$\left[\frac{1}{c}\frac{\partial}{\partial t} + \frac{\partial}{\partial z}\right]\epsilon(z,t) = 2\pi i\frac{\omega}{c}\int_{-\infty}^{t} dt'\chi(z,t,t')\epsilon(z,t'). \tag{11}$$

The solution are found via the Fourier representation:

$$\epsilon_{\text{out}}(L,t) = \int_{-\infty}^{\infty} \frac{d\omega}{2\pi} e^{-i\omega t} \epsilon_{\text{in}}(0,\omega) \exp\left[-2\pi i \frac{\omega}{c} \int_0^L dz \cdot \chi(\omega, z, \Delta_p, \Delta_c)\right], \quad (12)$$

where $\epsilon_{\text{in}}(z=0,\omega) = \int_{-\infty}^{\infty} dt e^{i\omega t} \epsilon_{\text{in}}(z=0,t)$ is the Fourier representation of the initial probe pulse, L is the length of the atomic medium with the optical depth (OD) $d_0 = n_0 \lambda^2 L$, and $\lambda = \lambda/2\pi$.

The transmission spectrum of the probe can thereby be expressed as:

$$T(\omega) = \exp\left[-4\pi \frac{\omega}{c} \text{Im}\left[\int_0^L dz \cdot \chi(\omega, z, \Delta_p, \Delta_c)\right]\right]. \quad (13)$$

The control-induced effective ground state decoherence leads to a reduction of the achievable transparency in the medium. Supplementary Figure 4 gives EIT spectra as a function of the probe detuning, for different ODs but for the same control power, i.e. the same effective decoherence rate. As can be seen, the maximal transmission achieved close to resonance decreases with the OD.

The overall loss during the propagation in the medium gives an upper bound η for this storage-and-retrieval efficiency. This bound is given by

$$\eta = \frac{\int_{-\infty}^{\infty} |\epsilon_{\text{out}}(z=L,t)|^2 dt}{\int_{-\infty}^{\infty} |\epsilon_{\text{in}}(z=0,t)|^2 dt}. \quad (14)$$

F. Decoherence during the storage duration

The finite lifetime of the memory leads to a decrease in the overall storage-and-retrieval efficiency. We give here this contribution in the case of residual magnetic fields [3].

Adiabatically switching off the control field coherently converts the probe field into a collective atomic excitation that can be written as:

$$|S(t)\rangle = \frac{1}{\sqrt{N}} \sum_{j=1}^{N} \sum_{m=-3}^{3} R_m e^{i\phi_{s_m g_m}(t)} |g_{1_m}, g_{2_m}, ..., g_{(j-1)_m}, s_{j_m}, g_{(j+1)_m}, ..., g_{N_m}\rangle. \quad (15)$$

The distribution of this collective excitation in the atomic medium depends on the polarizations of the probe and the control fields and it was analysed in details in [6]. The coefficients $R_m = C^{F_g,1,F_e}_{m,1,m+1}/C^{F_s,1,F_e}_{m,1,m+1}$ are ratio of Clebsch-Gordan coefficients for the probe and control fields [9, 10]. The magnetic field gradient for an atom in a state $|g_m\rangle$

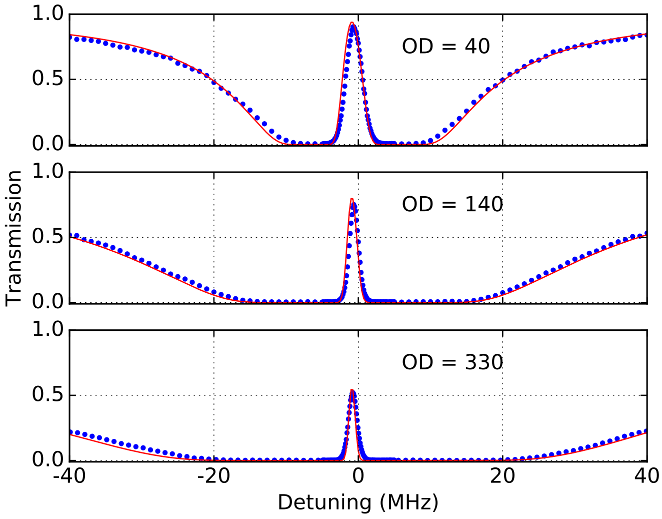

Supplementary Figure 4: EIT spectra for the Cs D_2 line as a function of the probe detuning for different OD when the control field power is kept constant (1 mW). The intrinsic ground state decoherence is $\gamma_0 = 10^{-3}\Gamma$ and the magnetic field gradient is set to $B_0 =$ 8mG.cm^{-1}, for a cloud length $L = 2.5$ cm. The red solid lines correspond to the present model, while the blue dots are experimental data.

Supplementary Figure 5: Storage-and-retrieval efficiency as a function of the OD using the Cs D_2 line. The brown line corresponds to one populated Zeeman sublevel in the ground state $|F_g = 3, m = 3\rangle$, while the blue line corresponds to equally populated Zeeman sublevels. The intrinsic ground state decoherence is $\gamma_0 = 10^{-3}\Gamma$ and the magnetic field gradient amplitude is $B_0 = 8$ mG.cm^{-1}, for a cloud length $L = 2.5$ cm. The gaussian probe pulse duration is $\tau = 0.5$ µs and the time delay is set to 2τ.

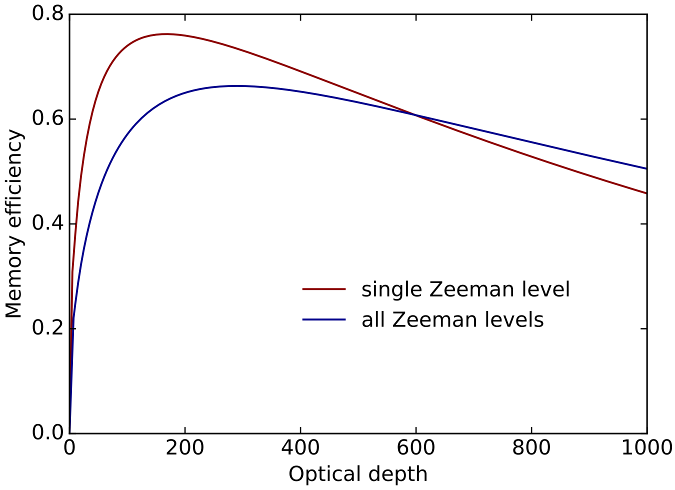

and at a position z over time t leads to a phase shift:

$$\Delta\phi_{s_m g_m}(z,t) = \frac{\mu_B m(g_{F_s} - g_{F_g})B(z)t}{\hbar}. \tag{16}$$

Assuming a distribution of the atomic density $n_0(z) = n_0 e^{-4z^2/L^2}$, the efficiency η_s due to the storage time is then evaluated as:

$$\eta_s \sim |\langle S(0)|S(t)\rangle|^2 = \left|\frac{1}{N}\sum_{m=-3}^{3} R_m^2 \int n(z)e^{i\Delta\phi_{s_m g_m}(z,t)}dz\right|^2 \sim \left|\sum_{m=-3}^{3} R_m^2 e^{-t^2/\tau_m^2}\right|^2, \tag{17}$$

where $\tau_m = \frac{2\sqrt{2}}{\mu_B m(g_{F_s} - g_{F_g})B_0 L}$.

G. Numerical simulation: overall storage and retrieval efficiency

As in the experiment, we consider a probe pulse with a Gaussian temporal profile and a FWHM duration τ as:

$$\epsilon_{\text{in}}(z=0,t) = \epsilon_0 \exp\left[-2\ln 2\frac{t^2}{\tau^2}\right]. \tag{18}$$

In the dynamic storage protocol, at time T_c the control pulse is turned off and the signal pulse is stored inside the atomic medium. Here, for each value of the OD, we choose the power of the control field to obtain a slow-light delay of the pulse $T_d = 2\tau$. In that case, in accordance with [8], it can be shown that the writing process has an efficiency close to unity for large OD (> 50), i.e. the leakage is negligible. However at low OD (< 50) the signal pulse cannot be contained entirely and a significant part of the pulse leaks from the medium before T_c. A strong leakage is usually observed and limits the efficiency. To take into account the leakage at low OD we change the lower limit of integration of the output pulse in (14) to T_c. The overall storage-and-retrieval efficiency can also be corrected by the finite efficiency η_s due to decoherence of the collective excitation during the storage time.

Supplementary Figure 5 provides the overall storage-and-retrieval efficiency as a function of OD. The effective ground state decoherence increases with the control field Rabi frequencies. This decoherence becomes more significant in the region of large optical depths due to the large control field power needed. This dependency leads to a reduction of the EIT transparency and, as a result, of the memory efficiency. In our configuration, without Zeeman pumping, the efficiency is limited to about 70%. The pumping of atoms to the edge state ($|g\rangle = |6S_{1/2}, F = 3, m = 3\rangle$) can lead to a slight increase in the memory efficiency. Our full model enables to compare the two implementations.

Supplementary References

[1] Carvalho, P. R. S., de Araujo, L. E. E. & Tabosa, J. W. R. Angular dependence of an electromagnetically induced transparency resonance in a Doppler-broadened atomic vapor. *Phys. Rev. A* **70,** 063818 (2004).
[2] Zhao, B. *et al.* A millisecond quantum memory for scalable quantum network. *Nat. Phys.* **5,** 95-99 (2009).
[3] Choi, K. S. Ph.D. thesis, California Institute of Technology, 2011.
[4] Mishina, O. S. *et al.* Electromagnetically induced transparency in an inhomogeneously broadened Λ transition with multiple excited levels. *Phys. Rev. A* **83,** 053809 (2011).
[5] Scherman, M., Mishina, O. S., Lombardi, P., Giacobino, E. & Laurat, J. Enhancing electromagnetically-induced transparency in a multilevel broadened medium. *Opt. Express* **20,** 4346 (2012).
[6] Sheremet, A. S. *et al.* Quantum memory for light via a stimulated off-resonant Raman process: Beyond the three-level Λ approximation. *Phys. Rev. A* **82,** 033838 (2010).
[7] Giner, L. *et al.* Experimental investigation of the transition between Autler-Townes splitting and electromagnetically-induced-transparency models. *Phys. Rev. A* **87,** 013823 (2013).
[8] Hsiao, Y.-F. *et al.* EIT-based photonic memory with near-unity storage efficiency. Preprint at http://arxiv.org/abs/1605.08519 (2016).
[9] Matsukevich, D. N., Chanelière, T., Jenkins, S. D., Lan, S.-Y., Kennedy, T. A. B. & Kuzmich, A. Observation of dark state polariton collapses and revivals. *Phys. Rev. Lett.* **96,** 033601 (2006).
[10] Tian, L., Li, S., Zhang, Z. & Wang, H. Suppressing decoherence of spin waves in a warm atomic vapor by applying a guiding magnetic field. *J. Phys. B* **48,** 035506 (2015).
